# Developmental Motor Profile in Preschool Children with Primary Stereotypic Movement Disorder

**DOI:** 10.1155/2019/1427294

**Published:** 2019-02-14

**Authors:** Francesca Valente, Chiara Pesola, Valentina Baglioni, Maria Teresa Giannini, Flavia Chiarotti, Barbara Caravale, Francesco Cardona

**Affiliations:** ^1^Department of Human Neurosciences, Sapienza University of Rome, Italy; ^2^Center for Behavioral Sciences and Mental Health, Istituto Superiore di Sanità, Rome, Italy; ^3^Department of Developmental and Social Psychology, Sapienza University of Rome, Italy

## Abstract

**Aim:**

Different neuropsychological dysfunctions have been described in children with primary Stereotypic Movement Disorder (SMD), mainly attention or motor coordination problems. Up to now with no study has evaluated psychomotor functions in preschoolers primary SMD. The aim of this observational study was to gather information on the motor profiles of SMD patients in this age range in comparison with typically developing children.

**Patients and Methods:**

Twenty-six children (four girls) aged 36 to 76 months (mean= 53 ±10) with primary SMD were assessed by a structured evaluation including the Movement Assessment Battery for Children-Second Edition (MABC-2), the Beery-Buktenica Developmental test of Visual-Motor Integration (VMI), the Repetitive Behaviour Scale-Revised (RBS-R), the Motor Severity Stereotypy Scale (MSSS), and the Child Behaviour Checklist (CBCL). The diagnoses of Intellectual Disability or Autism Spectrum Disorder were exclusion criteria from the study. A comparison group of twenty-seven (four girls) typically developing children without stereotypies aged 36 to 59 months (mean= 48 ±7) was also examined.

**Results:**

The MABC–2 total score was lower than 15th percentile in fifteen children with SMD (58%); the worst performances were observed in Balance and Manual Dexterity subtests. The motor coordination score of VMI was lower than 15th percentile in ten children (38%). The majority of the children with low scores at MABC-2 also had low scores at the motor coordination subscale of VMI. MABC-2 standard scores of the clinical group were significantly lower than those of controls on MABC-2 Total, Balance, and Ball Skills subtests.

**Conclusion:**

The finding of widespread dysfunction of gross and fine motor abilities in preschoolers with primary SMD seems to delineate a peculiar phenotype and could provide new approaches to the management of this neurodevelopment disorder.

## 1. Introduction

Stereotypies remain the most contentious issue among the repetitive behaviours in childhood, which also include tics, mannerisms, habits, compulsions, and other paroxysmal movements. Stereotypies are a pattern of repetitive nonfunctional motor behaviour that can interfere with the quality of social interaction, academic, or other activities or can result in injury [1]. Some practical criteria for differentiating the stereotypies from the other conditions have been proposed and largely accepted [2], but actually misdiagnoses are common, especially in nonspecialized settings [3]. Many other aspects of stereotypies have been debated in literature; even the current clinical definition and its usefulness as a categorical tool [4–6] have still not been firmly defined. Further, the functional basis of these movements, their aetiology, the possible underlying neuropathology, and, finally, their treatment, are still open questions [7–9].

The chief difficulty in dealing with stereotypies in childhood is related to their presence in different conditions: in Autistic Spectrum Disorders (ASD), in different genetic syndromes, in sensory impaired, or in developmentally disabled subjects as well as in typically developing children. Indeed, stereotypies often represent a physiological and transient finding, up to 60% of neurologically typical children showing some stereotypic movements or behaviours between 2 and 5 years [10].

Therefore, Stereotypic Movement Disorders (SMD) are classified as “primary”, indicating their presence in an otherwise typically developing child, or “secondary”, if another of the above-mentioned neuropsychiatric disorders is present.

Although children with primary SMD have a largely intact neurocognitive profile, motor problems that interfere with daily functioning have been reported. In particular, some previous studies have pointed up the relationship between stereotypies and Developmental Coordination Disorder (DCD) [11, 12] in school age children but none has specifically examined the preschoolers with SMD.

The aim of this study was to assess developmental motor profile in a group of preschool-aged children with primary SMD and to compare their motor characteristics with those of a group of typically developing subjects of the same age.

## 2. Methods

### 2.1. Participants

#### 2.1.1. Clinical Group

The clinical group consisted of 26 children (4 girls), aged 36 to 70 months (mean age = 53 months; SD=10) enrolled between January 2015 and July 2017 at the outpatient division of the Department of Human Neurosciences, Sapienza University of Rome, among those requesting a consultation for the presence of motor stereotypies.

The inclusion criteria for participation in the study were (1) presence of motor stereotypies (as defined by DSM 5^1^) evaluated by direct observation and/or by home videos; (2) preschool age; (3) adequate cognitive level (IQ> 70 evaluated by a standardized cognitive scale).

Exclusion criteria included (1) having a diagnosis of Autistic Spectrum Disorder, following the criteria of DSM 5^1^ and/or a comparison score higher than 4 at the Autism Diagnostic Observation Schedule-Second Edition (ADOS 2); (2) having other clinical neurological conditions, such as cerebral palsy or epilepsy, or other neuropsychiatric disorders, such as Attention Deficit and Hyperactivity Disorder, or neurosensorial impairments.

To verify the inclusion and exclusion criteria, children received a neurological, behavioural, and psychiatric assessment with standardized tests, including an evaluation of cognitive profile. The assessment was carried out by Medical Doctors expert in movement disorders and/or child psychiatry. In particular, at the Wechsler Preschool and Primary Scale of Intelligence (WPPSI-III) patients obtained a mean total IQ of 107 (SD = 12; range= 75-125). Questionnaires to check for other neuropsychiatric conditions (the Child Behaviour Checklist for ages 1^1/2^–5 or 4–18- CBCL) were also administered. The Motor Severity Stereotypy Scale (MSSS) and the Repetitive Behaviour Scale-Revised (RBS-R) were used to assess stereotypies.

#### 2.1.2. Control Group

The control group was recruited from two nursery/kindergartner public schools in Rome. Parents gave consent to participate in the study and completed a short questionnaire. The inclusion criteria for participation in the study were (1) the absence of motor stereotypies, reported from parents or evidenced by direct observation; (2) preschool age; (3) adequate cognitive level (IQ> 70 evaluated by a standardized cognitive scale). Exclusion criteria were a history of premature birth (being born before 37 weeks gestational age) and a history of neurodevelopmental, neurological, or congenital disorders. The control group included 27 children (4 girls) aged 36 to 59 months (mean= 48). All children had normal cognitive level evaluated by WPPSI-III (mean total IQ score=107; SD= 11; range= 74-125).

### 2.2. Procedures

All participants were evaluated for motor and visuomotor abilities by the Movement Assessment Battery for Children-Second Edition (MABC-2) and the Beery-Buktenica Developmental test of Visual-Motor Integration-6th Edition (VMI).

Children of clinical group were assessed during a three-day global evaluation including also language and behavioural abilities.

Children of the control group were assessed during regular school hours and, after completing the assessment, a feedback was given to parents about the findings.

The study was approved by the Ethic Committee of Sapienza University of Rome (Ref. 3477).

Parents gave their informed consent at the time of enrolment in the study.

### 2.3. Statistical Analysis

Quantitative data are summarized by means ± standard deviation (SD) and range, and categorical data by absolute and percent frequencies. In order to evaluate the effect of demographic and developmental variables on the severity of motor stereotypy, Mann-Whitney U test was used to analyse differences in MSSS and RBS-R scores between male and female patients, and between patients who crawled and patients who did not, or patients that attained walking after the age of 18 months in comparison with the remaining patients. The same tests were also used to compare patients to control children. To take into account the potential effect of age at assessment on the evaluation of the differences between patients and controls, we applied an analysis of covariance (ANCOVA), including group as independent factor and age at assessment as covariate.

Spearman nonparametric correlation coefficient was used to evaluate the association between age at assessment, age at attainment of walking, age at onset of repetitive movement, MSSS, RBS-R, MABC-2, and VMI scores.

Statistical analyses were performed by STATA Release 8.1.

## 3. Results

All children of the clinical group presented a normal neurologic examination (except for motor abnormalities, see below) and a normal cognitive profile.

The CBCLs showed mainly internalizing problems (2 patients with borderline scores and 7 in the clinical range); the more affected Symptoms Scales were Attention problems (6 and 2 patients, respectively), Withdrawn (2 and 3) and Somatic complaints (2 and 3). Within the DSM-oriented Scales, Pervasive Developmental problems (4 children with borderline and 4 with clinical scores) and ADHD problems (3 children with borderline and 1 with clinical scores) were the most affected ones (Table 1).

### 3.1. Stereotypies Evaluation

The onset of stereotypies was reported at a mean age of 19 months (SD: 13; range 4-51 months). Their semiology had remained unchanged over time, mostly characterized by complex motor stereotypies: patients presented a single repetitive movement or complex sequences involving the entire body such as jumping, kicking, flapping hands, moving hands in front of the face or the eyes, or involving movements and “dystonic” postures of the trunk. Sounds or vocalizations accompanied the motor stereotypies in 4 patients. The frequency of stereotypies has been reported to increase from the time of their onset. Commonly described triggers were excitement or boredom.

The MSSS showed that each child had a limited number of stereotypies, comprised between 1 and 3 (mean: 1.6); their frequency and intensity were mild (range 1-4 for both; mean: 2.6 and 2.8 respectively); the interference of stereotypies was variable, from 0 to 4 (mean: 1.7). The mean MSSS final score was 20 (SD =11; range= 4-53), suggestive of a mild impairment in the daily life.

At the RBS-R, items of the subscale of “Stereotypic behaviours” were positive in all children; moreover, the questionnaire revealed the presence of other repetitive behaviours in several children, mainly “Ritualistic behaviour” and “Sameness behaviour”, even if at a lower degree (Table 2). The mean Global Rating score was of 31.6, in a scale from 1 to 100, with a wide range between 1 and 80.

No significant differences in stereotypies severity were observed between subgroups of children (males versus females, children who crawled versus children who did not).

### 3.2. Motor Skills

Children with stereotypies had a delay in achieving motor milestones: walking alone was at a mean age of 14.6 months (range: 11-21) and in 5/27 (18 %) it was achieved after the age of 18 months; moreover 12 out of 26 children (46%) skipped the crawling stage.

Neurological evaluation revealed difficulties in coordination tasks and clumsiness in all patients.

In 15 patients (58%) the MABC–2 total score was below the 15th percentile and in 4 of them (15%) below the 5th percentile. The worst performances were observed in the tasks of Balance and Manual Dexterity, with relatively better results in Ball Skills.

At the VMI, 10 children (38%) obtained scores below the 15th percentile in the motor coordination subscale, while the visual perception and visual-motor integration scores were in the normal range in all but 3 and 2 cases, respectively.

In all but six cases the children with low scores at MABC-2 also had low scores at the motor coordination subscale of VMI.

MABC-2 total and manual dexterity scores were significantly lower in males than in females (Mann-Whitney: p=0.0488 and p= 0.0112, respectively) and in patients that attained walking after the age of 18 months in comparison with the remaining group (Kruskal-Wallis test: p=0.0059; Fisher's exact: 0.034 and Kruskal-Wallis test: p=0.0044; Fisher's exact: 0.01, respectively). Moreover, a significant negative correlation was found between age of walking and MABC-2 Total (Spearman r_s_= -0.4722, p= 0.0149), Manual dexterity (Spearman r_s_= -0.4717, p= 0.0332), and Balance (Spearman r_s_= -0.4792, p= 0.0133) scores.

No other significant differences in motor skills were observed between subgroups of patients (children who crawled versus children who did not) neither significant correlation with clinical variables (age of stereotypies onset, MSSS, and RBS scores and subscores).

### 3.3. Comparison with Control Group

Patients and controls were matched for sex and age (Table 3).

The MABC-2 standard scores of the clinical group were significantly lower than those of control group on MABC-2 Total, Balance, and Ball Skills subtests (Table 3). Moreover, the distribution of the MABC-2 Total percentile scores (>15th percentile, 5th-15th percentile, <5th percentile) was significantly different between patients and controls, the formers showing a higher rate of low scores (Mann-Whitney: p=0.0303).

On the other hand, controls had significantly lower scores than patients on VMI Visual subtest (Table 3). Finally, no differences between and controls were found in VMI total and VMI Motor scores.

These differences were confirmed in ANCOVA. As expected, the covariate age at assessment significantly affected scores related to motor coordination (MABC Ball Skills p=0.0036, and VMI Motor p=0.0276), but its introduction in the model did not affect the significance of the differences between SDM patients and controls.

## 4. Discussion

In our sample of preschoolers with primary SMD we found widespread difficulties in the gross and fine motor abilities. Indeed, in 58 % of the cases we observed motor difficulties during a number of coordination tasks, mainly in the Balance and Manual Dexterity subtests. The executive nature of these difficulties was confirmed by the absence of involvement of visual perceptive processes (VMI). Moreover, these findings seem to be in line with the anamnestic data indicating an atypical (several children had never crawled) and often delayed motor development.

Some authors have already described the presence of motor impairment in children with SMD [3, 12, 13], but no study has focused on the preschool age. In the SMD series of Mahone et al. [12] (range age: 4–12 years) one-third of children were rated at the DCDQ as having motor skill difficulties consistent with DCD. In the Freeman's study [3], DCD was one of the more frequent comorbidity (38%) in a sample of typically developing children with primary SMD aged between 3 and 9 years.

The reason for the higher rate of motor abnormalities we have found in comparison with other studies is not clear. Obviously, a sampling bias cannot be excluded: even if the presence of stereotypies was the principal reason for referral, the cooccurrence of stereotypic behaviours and motor difficulties could have induced parents to ask for a consultation. Otherwise, the assessment of children by structured tests could have allowed us to discover some minor abnormalities, which may not have been noticed or rated in the questionnaires compiled by parents. Finally, the younger age of our patients in comparison with previous reports, conducted with groups of school age children, could have accounted for this higher rate. In this regard, contrasting data have been reported on the permanency of motor difficulties observed in preschool-aged children. In a population-based study on about 3000 preschoolers by a short motor screening test Pless et al. [14] found that motor status of children with poor motor performance at the age of 5 and 6 years had not changed by the time of the successive follow-up. These data were not replicated by Waelvelde et al. [15]: in a clinically referred sample of children with or at risk for ASD, ADHD, and/or DCD, with a follow-up at different age, they found an improvement of motor performances, but for the ASD group in which the motor problems were more stable. The cross-sectional design of our study does not allow us to say if the motor abnormalities we have found will be stable in the long term or they will improve during school age or adolescence; only prospective studies could address this important issue.

Obviously, the high prevalence of impaired motor skills among young SMD patients raises the question of a possible pathogenic relationship between repetitive movements and motor dysfunctions. In this regard, two different aspects can be highlighted.

Firstly, although the pathophysiologic mechanism responsible for stereotypies has not been fully clarified, the pathobiological hypotheses have pointed to an abnormality in the motor control within corticostriatal-thalamo-cortical (CSTC) circuits, with a possible involvement of the habit-related pathways, from the supplemental motor area to the putamen [16]. Available clinical and animal model data have suggested that the sensorimotor loop is primarily involved in abnormal stereotypical motor behaviour [17]. Further support for basal ganglia involvement in stereotypies has come both from volumetric imaging data indicating a decreased volume of the putamen-caudate [18, 19] and from MR spectroscopy results showing lower levels of GABA in the anterior cingulate cortex and striatum of subjects with primary SMD [20]. Recently, Houdayer et al. [21] suggested that primary motor stereotypies could be due to a motor command release independent from the CSTC motor loops usually involved in voluntary motor control. This motor command would most probably originate from the basal ganglia and activate a different pattern of sensorimotor loops. In agreement with these hypotheses, Mahone et al. [12] proposed that the motor coordination abnormalities among children with primary SMD could be related to a dysfunction within the CSTC circuits or in contributing regions, such as the cerebellum, which have direct connections to the CSTC structures.

On the other hand, some studies have reported the presence of motor developmental delay and impairment in children with secondary stereotypies [22]. In particular abnormalities in gait and balance, slower speed of timed movements, coordination problems and greater “overflow” movements have been observed in children with ASD [23]. Different degrees of motor impairments, with difficulties in the coordination ability, delay in fine and gross motor development and/or presence of other abnormal movements, have also been reported in other disorders in which repetitive movements or behaviours represent a pivotal symptom, such as Tourette Syndrome (even if with inconsistent results [24, 25]) or Obsessive-Compulsive Disorder [26]. Thus, these studies seem to delineate a cluster of motor symptoms common to different neurodevelopmental disorders, which need to be further investigated.

The repetitive movements presented by our patients were characterized by complex motor stereotypies, most frequently involving the arms, the trunk, and the mouth, but rarely accompanied by vocalizations. Their frequency has been reported to increase since their onset, as already described in primary SMDs [27]. Notwithstanding the DSM-5 based diagnosis of ASD being an exclusionary criterion for this study, several children showed, even if at a lower degree than stereotypies, some autistic traits, such as sameness, ritualistic, or compulsive behaviours, and tendency to be withdrawn; together with the nonspecific patterns of stereotypies, these findings underline the challenge of the categorical diagnosis of motor stereotypies [28] and the need for further studies.

### 4.1. Limitations

Some limitations to this study need to be considered. A first limitation pertains to the small sample size; however, we have also observed the same clinical characteristics (delay in motor achievements; poor motor skills) in a larger series of younger and older children with primary SMD (data not shown). The difference of age (statistically not significant) between patients and controls could constitute another limitation; however, in this regard, the relatively younger age of the controls strengthens the value of the motor abnormalities we have found in patients with SMD. Finally, in our sample there is a clear prevalence of males, but this reflects the prevalence reported for SMD patients.

## 5. Conclusions

In spite of the above-mentioned limitations, this study confirms that children with primary SMD show a widespread dysfunction of gross and fine motor abilities and highlights that this may be evidenced even in preschool age. This observation could delineate a peculiar phenotype and could provide new approaches to the management of this neurodevelopmental disorder. In particular, SMD preschoolers showing motor dysfunctions could take advantage from treatments for improving their motor abilities. This in turn could reduce the lack of self-esteem and the tendency to withdrawn that characterize children with DCD and consequently attenuate some triggers of stereotypies (isolation; boredom).

Further studies, in different ages of development, are needed to extend this preliminary description in order to investigate the longitudinal trajectory and a more specific neuropsychological and neuromotor profile of children with primary SMD.

## Figures and Tables

**Table 1:** Number and percentage of children with SMD obtaining borderline or clinical scores at the Children Behaviour Checklist (CBCL)-ages 1.5–5 or 4–18, according to chronological age.

	Borderline	Clinical
CBCL Total	2 (8%)	6 (23%)

CBCL Internalizing problems	2 (8%)	7 (27%)

CBCL Externalizing problems	3 (11%)	5 (19%)

Symptoms scales		

Emotionally reactive	3 (11%)	1 (4%)

Anxious/depressed	0 (0%)	2 (8%)

Somatic complaints	3 (11%)	2 (8%)

Withdrawn	3 (11%)	2 (8%)

Sleep problems	0 (0%)	1 (4%)

Attention problems	2 (8%)	5 (19%)

Aggressive behavior	2 (8%)	0 (0%)

DSM-oriented scales		

Affective problems	2 (8%)	0 (0%)

Anxiety problems	1 (4%)	1 (4%)

Pervasive Developmental problems	4 (15%)	4 (15%)

ADHD problems	0 (0%)	3 (11%)

Oppositional Defiant problems	1 (4%)	0 (0%)

**Table 2:** Repetitive Behaviour Scale-Revised (RBS-R): subscale scores and number endorsed in children with SMD.

	Subscale Scores (Mean ± SD)	Number Endorsed*∗* (Mean ± SD)	Number and percentage of patients with number endorsed ≠0
I- Stereotypic behavior	3.9 ± 2.4	2.1 ± 2.1	26 (100%)

II- Self-injurious behavior	0.2 ± 0.5	0.2 ± 0.5	5 (19%)

III-Compulsive behavior	2.0 ± 3.7	1.1 ± 1.7	12 (46%)

IV-Ritualistic behavior	2.0 ± 2.7	1.3 ± 1.5	14 (54%)

V- Sameness behavior	3.2 ± 3.9	2.3 ± 2.1	20 (77%)

VI-Restricted behavior	1.5 ± 1.6	0.9 ± 0.8	17 (65%)

*∗*Number of items endorsed for each subscale (any rating other than zero).

**Table 3:** Comparison between SMD patients and controls.

	SMD patients	Controls	
M/F ratio	22/4	23/4	Fisher' exact= 1.000

Mean age at evaluation (months)	52.8	48.8	§p= 0.0624

MABC Total*∗*	7.1 ± 3.3	8.7 ± 2.7	§p= 0.0303 #p= 0.0854

MABC Manual dexterity*∗*	7.± 3.6	7.1 ± 3.2	§p= 0.7744 #p= 0.6913

MABC Balance*∗*	7.3 ± 3.1	10.3 ± 2.5	§p= 0.0001 #p= 0.0007

MABC Ball skills*∗*	9.0 ± 3.7	13.5 ± 5.9	§p= 0.0038 #p= 0.0075

VMI Total*∗*	99.2 ± 14.3	104.8 ± 10.9	§p= 0.1281 #p= 0.2235

VMI Visual*∗*	108.1 ± 16.0	98.3 ± 14.7	§p= 0.0215 #p= 0.0187

VMI Motor*∗*	86.8± 15.6	89.0 ± 15.4	§p= 0.7849 #p= 0.1985

*∗*Standard scores.

§Mann-Whitney.

#ANCOVA with group (SMD patients versus controls) as grouping factors and age at assessment as covariate; p refers to the effect of group.

## Data Availability

The clinical data used to support the findings of this study are available from the corresponding author upon request.
